# Brain Metastasis in Pediatric Patients with Osteosarcoma

**DOI:** 10.3390/curroncol31110516

**Published:** 2024-11-09

**Authors:** Jacob Murphy, R. Taylor Sundby, Erin E. Resch, Ruyan Rahnama, Kathryn M. Lemberg, Alexandre Maalouf, Aditya Suru, Jason Fixler, Brian H. Ladle, Daniel S. Rhee, Adam S. Levin, Aparna Pallavajjala, Christopher Gocke, Matthew M. Ladra, Mari L. Groves, Sahaja Acharya, John M. Gross, Nicolas J. Llosa, Christine A. Pratilas

**Affiliations:** 1 Johns Hopkins University School of Medicine, Baltimore, MD 21287, USA; jmurp103@jhmi.edu; 2 Pediatric Oncology Branch, Center for Cancer Research, National Cancer Institute, National Institutes of Health, Bethesda, MD 20892, USA; taylor.sundby@nih.gov; 3 Division of Pediatric Oncology, Department of Oncology, The Sidney Kimmel Comprehensive Cancer Center, Johns Hopkins University School of Medicine, Baltimore, MD 21287, USA; eresch3@jhmi.edu (E.E.R.); rrahnam1@jhmi.edu (R.R.); klember1@jhmi.edu (K.M.L.); amaalou1@jhmi.edu (A.M.); bladle@jhmi.edu (B.H.L.); nllosa1@jhmi.edu (N.J.L.); 4 Department of Chemical and Biomolecular Engineering, Johns Hopkins University, Baltimore, MD 21218, USA; asuru1@jhu.edu; 5 Division of Pediatric Hematology-Oncology, Sinai Hospital of Baltimore, Baltimore, MD 21215, USA; jfixler@lifebridgehealth.org; 6 Department of General Pediatric Surgery, Johns Hopkins University, Baltimore, MD 21218, USA; 7 Department of Orthopedic Surgery, Johns Hopkins University School of Medicine, Baltimore, MD 21287, USA; alevin25@jhmi.edu; 8 Department of Pathology, Johns Hopkins University School of Medicine, Baltimore, MD 21287, USA; apallav2@jhmi.edu (A.P.); cgocke1@jhmi.edu (C.G.); jgross28@jhmi.edu (J.M.G.); 9 Department of Radiation Oncology, Johns Hopkins University School of Medicine, Baltimore, MD 21287, USA; mladra@jhmi.edu (M.M.L.); sachary7@jhmi.edu (S.A.); 10 Department of Neurosurgery, Johns Hopkins University School of Medicine, Baltimore, MD 21287, USA; mgroves2@jhmi.edu

**Keywords:** osteosarcoma, brain metastasis, pediatric oncology

## Abstract

Background: Brain metastases in pediatric osteosarcoma are infrequent but associated with a dire prognosis. Methods: This retrospective study examined six pediatric patients at Johns Hopkins Hospital who developed brain metastases from osteosarcoma between April 2015 and November 2023. Results: Median survival post-brain metastasis was 2.5 months. The patients underwent various treatments, including chemotherapy, surgery, and radiation. Despite these interventions, outcomes were uniformly fatal. Notably, one patient survived over 13 months post-brain metastasis with a treatment regimen of cabozantinib and nivolumab along with surgical resection and radiation, highlighting the potential efficacy of multimodal treatment regimens. This case demonstrated changes in the immune microenvironment, hinting at an anti-tumoral response, although no histologic response was observed. Conclusions: These findings emphasize the critical need for vigilant clinical monitoring, especially in patients with new neurological symptoms. The study highlights the diagnostic challenges and the rapid progression of brain metastases, underscoring the necessity for further research. Prospective studies and clinical trials focusing on novel therapeutic strategies are essential to improve outcomes. Disease biology studies examining tumor features across primary, pulmonary, and brain metastatic sites may offer insights into the mechanisms of metastasis and potential therapeutic targets, providing a foundation for better management of this devastating complication.

## 1. Introduction

Brain metastases are rare events in pediatric solid malignancies but portend a poor prognosis. While many pediatric malignancies, including neuroblastomas, pleuropulmonary blastomas, germ cell tumors, and nephroblastomas, may metastasize to the brain, sarcomas are the most common source of brain metastasis among pediatric patients, with an estimated incidence of 5–6% [1,2,3,4]. Though the mechanism responsible for this predilection remains unclear, pulmonary metastatic disease has been postulated as the most significant risk, and in published reports, pulmonary metastases precede brain metastasis in 60–85% of cases [5,6,7]. Brain metastasis in patients with sarcoma has a near-universally fatal outcome and, therefore, remains a critical area warranting investigation.

Median post-brain metastasis survival is reported to be approximately two months [3,4]. Patients are often treated with multimodal therapy consisting of combination chemotherapy and local control with surgery and/or radiation therapy where feasible. However, despite recent advances in the biology and treatment of osteosarcoma and a recognized incidence of brain metastases, there is no consensus treatment regimen, nor has it been possible to study treatment for such patients using prospective or randomized approaches [3,8,9].

Here, we aim to contribute to the body of knowledge regarding diagnosis, treatment, clinical course, and outcomes for these patients. We describe a retrospective cohort summary of six patients with osteosarcoma who developed brain metastasis, including one patient who achieved survival of over 13 months with multimodal therapy.

## 2. Patients and Methods

This retrospective electronic medical record study evaluated fifty-one patients with osteosarcoma treated at Johns Hopkins Hospital from April 2015 to November 2023. From this cohort, patients were selected for further analysis if they had a pathologic diagnosis of osteosarcoma, developed brain metastases, and received all or part of their treatment in the Division of Pediatric Oncology at JHH. We identified six pediatric patients meeting these criteria. When available, tissue from the primary tumor and metastases underwent targeted next-generation sequencing (NGS) with the Johns Hopkins NGS Solid Tumor Panel or Foundation One Heme test (https://www.foundationmedicine.com/test/foundationone-heme (accessed on 15 July 2024)) in Clinical Laboratory Improvement Amendments (CLIA) certified laboratories.

Time to progression (TTP) was defined as time from pathologically confirmed osteosarcoma to first evidence of radiologic progression. Overall survival (OS) was measured from the time of pathologic diagnosis to the date of death or the most recent encounter with our health system. Progression-free survival (PFS) was defined as the time of pathologic diagnosis to the date of first progression or the most recent encounter with our health system. The institutional review board (IRB) of Johns Hopkins University approved this retrospective clinical data extraction and analysis.

## 3. Results

Six patients were identified who had osteosarcoma and experienced brain metastasis. Clinical summaries are provided in Table 1.

### 3.1. Patient #1

**Figure 1:**
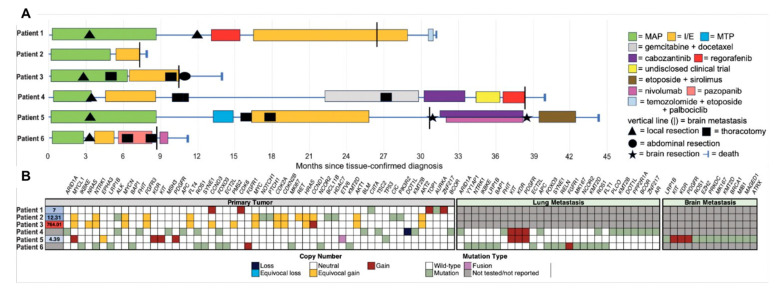
The clinical courses and genomic characteristics of six patients with osteosarcoma brain metastases. (**A**) Swimmer’s plot depicts the treatment course and clinical histories; (**B**) the mutational landscape of primary osteosarcoma following neoadjuvant chemotherapy and, when available, lung and brain metastases. Clinical sequencing results were derived from the Johns Hopkins NGS Solid Tumor Panel and Foundation One Heme assay. I/E, ifosfamide/etoposide; MAP, methotrexate, cisplatin, and doxorubicin; MTP, muramyl tripeptide.

**Figure 2:**
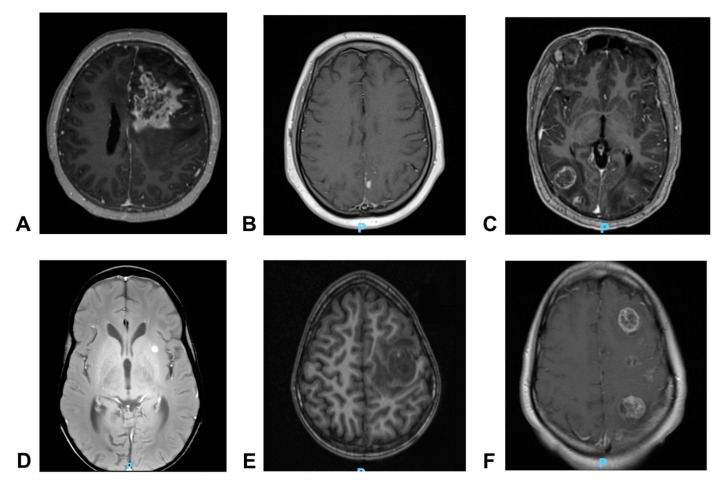
Representative axial T1 MRI imaging initial brain metastases of (**A**) Patient 1; (**B**) Patient 2; (**C**) Patient 3; (**D**) Patient 4; (**E**) Patient 5; and (**F**) Patient 6.

Patient #1 was an 8-year-old male with localized, high-grade osteoblastic osteosarcoma of the left distal femur. He received treatment per Children’s Oncology Group (COG) protocol AOST0331 with neoadjuvant and adjuvant methotrexate, cisplatin, and doxorubicin (MAP) and local control with a joint-sparing left femur resection (Figure 1A). Resection demonstrated 85% tumor necrosis with negative margins. Four months post-operatively, (18F) fluorodeoxyglucose (FDG) positron emission tomography (PET)/computed tomography (CT) identified intense avidity within the musculature of the left medial thigh, subsequently confirmed as locally recurrent osteosarcoma. The patient proceeded with hip disarticulation followed by adjuvant regorafenib for nearly three months but experienced progression with new lung, spine, and surgical site soft tissue disease. Ifosfamide/etoposide was tolerated for 12 cycles before severe headaches prompted brain magnetic resonance imaging (MRI), which identified a 4.7 cm mass within the left frontal lobe and a 2.6 cm mass within the right temporal lobe (Figure 2A). Three additional cycles of ifosfamide/etoposide (I/E) were then given concurrently with palliative radiation. One month later, the patient presented with new-onset tonic–clonic seizures, and MRI revealed an increase in the size of the left frontal lobe mass with a new right midline shift of 8 mm. The brain lesions continued to grow, causing altered mental status (Figure 1A). The patient was treated with one cycle of temozolomide, etoposide, and palbociclib (chosen based on CCND3 copy number gain) with concurrent palliative radiation to the same radiation field, but the patient died two weeks later from progressive neurologic decline, 5 months after identification of CNS metastatic disease.

### 3.2. Patient #2

Patient #2 was a 20-year-old male with localized, grade 3 chondroblastic osteosarcoma of the L5 vertebral body and sacrum. He started MAP chemotherapy, and imaging demonstrated radiographic disease stability, but the tumor was deemed unresectable without substantial morbidity. To estimate response to therapy in an otherwise unresectable tumor, a biopsy was performed, which demonstrated no evidence of tumor necrosis. The patient continued MAP therapy for three more cycles until re-staging scans revealed interval growth of the sacral tumor. Treatment was transitioned to high-dose I/E (Figure 1A). Following two cycles of I/E, the patient experienced a cluster of seizures, and brain MRI identified intracranial metastatic disease with an extra-axial enhancing mass in the high left parietal convexity and leptomeningeal enhancement in the left parietal sulci (Figure 2B). Chest CT concurrently identified multiple pulmonary nodules consistent with metastatic disease. The patient was discharged home with hospice support and died suddenly from acute cardiorespiratory collapse 11 days after identification of central nervous system (CNS) metastatic disease.

### 3.3. Patient #3

Patient #3 was a 16-year-old male with grade 3 osteoblastic osteosarcoma of the right distal femur with pulmonary and osseous metastases (more than 15 pulmonary nodules and a 1.6 cm left parasagittal parietal bone lesion). The patient received MAP chemotherapy with distal femur resection after two cycles, revealing negative margins and 55% necrosis. Subsequent right-sided thoracotomy identified and resected 61 pulmonary nodules. Re-staging imaging after four cycles of MAP revealed radiographic on-therapy progression of the remaining pulmonary disease, and chemotherapy was changed to I/E (Figure 1A). He completed four cycles of I/E before undergoing a planned left-sided thoracotomy with lower lobe lobectomy, but post-operative imaging revealed further progression of bilateral pulmonary metastases and new subcutaneous nodules in the anterior abdominal wall. Two weeks later, he developed severe headaches, nausea, vomiting, and blurry vision, prompting a brain MRI, which revealed numerous newly appearing supratentorial and infratentorial rim-enhancing metastatic lesions in the parietal–occipital lobes, left internal capsule, and bilateral cerebellar hemispheres (Figure 2C). Whole-brain radiation was delivered at a palliative dose (30 Gy, 3D conformational radiation, 10 fractions). He developed new-onset seizures, and CT of the head revealed interval enlargement of the multiple left-sided cerebral metastases. An MRI then revealed new enlarging lesions and worsening perilesional vasogenic edema with effacement of the fourth ventricle. His subsequent course was complicated by status epilepticus, and he died 3 months after the identification of parenchymal brain metastases.

### 3.4. Patient #4

Patient #4 was an 11-year-old male with a known germline *RB1* mutation and a history of metastatic retinoblastoma who developed grade 3 osteoblastic osteosarcoma of the right proximal tibia with numerous bilateral lung metastases. The patient had been previously treated for metastatic retinoblastoma with radiation therapy, chemotherapy (etoposide, vincristine, carboplatin), and consolidation with autologous bone marrow transplant four years prior to identification of osteosarcoma. He received two cycles of MAP prior to resection of the right proximal tibia mass, which demonstrated 50% necrosis and negative margins. Given prior chemotherapy exposure, he then transitioned to receive five cycles of adjuvant I/E, but therapy was discontinued due to complications of posterior reversible encephalopathy syndrome (PRES) and status epilepticus. An MRI of the brain obtained in the setting of PRES demonstrated no evidence of intracranial disease.

After recovery from PRES, the patient underwent staged bilateral thoracotomies, with 14 osteosarcoma metastases removed from the left lung and 11 from the right (Figure 1A), and remained without radiographic evidence of osteosarcoma for 11 months. He then developed a mass and swelling over the right zygomatic arch, and an MRI revealed a new 6.3 cm mass in the right maxillary sinus. Biopsy revealed an undifferentiated pleomorphic sarcoma; simultaneously, CT chest revealed new pulmonary nodules, which were later biopsy proven to be metastatic osteosarcoma. He was treated with four cycles of gemcitabine and docetaxel, radiation (50 Gy, intensity-modulated radiation therapy (IMRT), 20 fractions) to the maxillary sinus mass and a repeat left-sided thoracotomy with resection of pulmonary metastases. One month after the thoracotomy, CT again revealed progression of right-sided pulmonary metastases.

The patient was enrolled in a clinical trial and treated with cabozantinib [10], and a CT demonstrated a radiographic response of lung metastases after one cycle. After four total cycles, however, chest CT again revealed progression of pulmonary metastases. He then enrolled in a new clinical trial but again experienced the progression of pulmonary metastases after two cycles. Regorafenib was started but discontinued after two cycles for further progression, and surveillance MRI of the face/orbits revealed multiple new asymptomatic lesions within the left putamen measuring 0.6 cm, right occipital lobe juxtacortical white matter measuring 0.4 cm, right medial temporal lobe measuring 0.4 cm, and a small focus in the right parietal lobe (Figure 2D). A biopsy of intracranial lesions was not performed, and it remained unknown if the intracranial metastases originated from the osteosarcoma or the undifferentiated pleomorphic sarcoma. Disease-directed therapy was discontinued, and the patient died two months later.

### 3.5. Patient #5

Patient #5 was a 9-year-old male with localized, high-grade osteosarcoma of the left distal femur, treated with standard MAP neoadjuvant chemotherapy. Resection demonstrated 90% tumor necrosis and negative margins. Six months after completion of adjuvant MAP, surveillance imaging detected bilateral pulmonary nodules. I/E salvage therapy was started after the patient underwent the first of two staged bilateral thoracotomies and was continued for a total of 12 cycles (Figure 1A).

**Figure 3:**
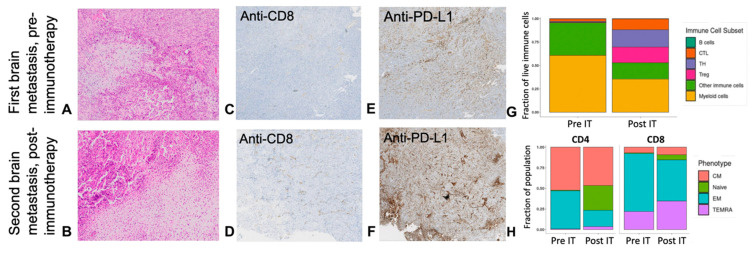
Histology, immunohistochemistry (IHC), and flow cytometry of brain metastases in Patient 5 pre- and post-immunotherapy (IT). (**A**,**B**) Histologic sections 100x of pre (**A**) and post (**B**) are morphologically similar and display high-grade conventional osteosarcoma, showing sheets of mitotically active tumor cells producing osteoid and chondroid matrix, respectively. Significant histologic response to therapy is not identified. (**C**,**D**) Pre- and post-immunotherapy photomicrographs showing anti-CD8 IHC stains at 4× magnification. (**E**,**F**) Pre- and post-immunotherapy photomicrographs displaying anti-PD-L1 IHC stains at 4× magnification; (**G**) Stacked bar chart showing the proportion of the different subsets of freshly isolated tumor-infiltrating immune cells characterized via multiparameter flow cytometry from tumor specimens obtained pre- (pre-IT) and post- (post-IT) immunotherapy. B cells: B lymphocytes; CTL: cytotoxic T cells; TH: helper T cells; Treg: regulatory T cells. (**H**) Stacked bar chart depicting the proportion of intra-tumoral memory T cell compartments according to defined phenotypes assessed by multiparameter flow cytometry pre-IT and post-IT. CM: central memory T cells, Naïve: naïve T cells; EM: effector memory T cells; TEMRA: terminally differentiated effector memory T cells re-expressing CD45RA.

Four months after I/E completion, surveillance whole body positron PET/CT imaging again detected pulmonary nodules and identified a new asymptomatic FDG avid brain lesion. Brain MRI confirmed an enhancing mass in the left posterior frontal lobe with cystic and necrotic components measuring 2.7 cm abutting the left ventricle (Figure 2E). The brain lesion was resected, the resection cavity was radiated (24 Gy, stereotactic radiation, three fractions), and the patient began therapy with cabozantinib and nivolumab. Histologic sampling of the brain lesion identified metastatic high-grade conventional osteosarcoma (Figure 3A). Stable disease was achieved for 7 months before the identification of new pulmonary nodules and intracranial disease recurrence. A second subtotal neurosurgical resection confirmed morphologically similar high-grade conventional osteosarcoma without histologic evidence of treatment response (Figure 3B). Interrogation of the tumor microenvironment of tumor specimens procured pre- and post-combination therapy revealed a higher intra-tumoral infiltration of CD8+ T cells (Figure 3C,D) and a higher expression of PD-L1 via immunohistochemistry (Figure 3E,F). Furthermore, multiparameter flow cytometry studies of freshly isolated tumor-infiltrating lymphocytes from specimens demonstrated the expansion and contractions of relevant subsets of immune cells following anti-PD1 directed therapy. The latter was characterized by an increase in the proportion of anti-tumoral helper T cells and cytotoxic T cells, along with a contraction of the immunosuppressive myeloid cells (Figure 3G). Additionally, the main intra-tumoral T cell profiles were compatible with effector memory T cell and terminal effector memory T cell phenotypes in the CD8+ T cell compartment, potentially indicating their cytotoxic activity against cancer cells and contributing to anti-tumor immune responses despite a lack of histologic response to therapy (Figure 3H). Upon progression, the patient was treated with radiation (30 Gy, stereotactic radiation, five fractions) and palliative oral chemotherapy with etoposide and sirolimus. Repeat imaging ultimately revealed further intracranial disease progression with midline shift; the patient transitioned to end-of-life care and died 1.5 months later, 13 months after the identification of CNS metastatic disease.

### 3.6. Patient #6

Patient #6 was a 17-year-old female with high-grade chondroblastic osteosarcoma of the right femoral diaphysis with bilateral pulmonary metastases, also treated with standard MAP chemotherapy. Resection of the right femoral mass after two cycles demonstrated 10–20% necrosis and negative margins. Post-resection chest CT revealed pulmonary disease progression, prompting a change in therapy to high-dose I/E. The patient experienced further progression of pulmonary disease after two cycles. She was next treated with three cycles of pazopanib with concurrent staged bilateral thoracotomies for resection of lung metastases (23 nodules resected on the left side, then 11 nodules resected on the right side) (Figure 1A).

One week following the right-sided (second) thoracotomy, the patient developed right-sided facial numbness, right-sided weakness in the distribution of the facial nerve, and right-sided gaze preference. Brain MRI revealed 10 enhancing and hemorrhagic lesions within the left supratentorial cerebral hemisphere measuring up to 3 cm, and PET/CT detected new hepatic and left lower lobe pulmonary metastases (Figure 2F). Whole brain radiation (30 Gy in 10 fractions + 15 Gy boost in five fractions to largest regions of gross disease, 3D conformal) was delivered, and she was treated with a single dose of nivolumab. Hepatic disease progression ensued quickly and was accompanied by new splenic and pulmonary lesions. The patient elected for no further cancer-directed therapy, and she died 1.5 months later, 2.5 months following identification of CNS metastatic disease.

## 4. Discussion

Brain metastases are an infrequent but universally fatal complication in osteosarcoma. A lack of prospective studies and a paucity of research leaves clinicians ill-equipped to predict or manage this complication. This retrospective analysis contributes to the fund of reported clinical experiences managing brain metastases in osteosarcoma. More specifically, our limited cohort describes a wide range of presentations, including one patient who achieved survival for over one year after developing brain metastasis and one patient who experienced rapid development of substantial intra-cranial disease despite recent surveillance imaging without any findings.

Five out of six patients in our cohort developed brain metastasis after pulmonary disease, while one patient developed intracranial and pulmonary metastases concurrently. Interestingly, the latter was the only patient not to undergo resection of the primary tumor. Additionally, this patient had a primary tumor located in the axial skeleton. While brain metastases are typically thought to originate from lung metastases, it is possible that the potential for intracranial disease to arise directly from the primary tumor site, particularly when the primary tumor is in the axial skeleton, is underappreciated. The rarity of brain metastases in osteosarcoma confounds our understanding of factors that contribute to the development of brain metastases, such as underlying genetic mutations driving individual tumor biology. No obvious relationships between metastases and contributing genomic risk factors could be discerned from our cohort of patients (Figure 2B).

While all patients in our cohort experienced fatal outcomes, consistent with outcomes reported in the literature, Patient 5 experienced a unique post-brain metastasis survival of 402 days (more than 13 months). This patient would not have been identified de novo as a high risk for poor outcome, given localized disease at diagnosis and a “good” chemotherapy response (90% necrosis at time of resection), however, first relapse was detected less than one year from completion of upfront treatment. He developed a solitary left frontal lobe metastasis 31 months after initial diagnosis, which was resected and radiated. Stable disease was achieved for over 7 months with a regimen of cabozantinib and nivolumab. Consistent with prior reports, this patient’s prolonged survival following brain metastasis may be attributable to the clinical features of a solitary lesion and treatment with multi-modal therapy, including resection and radiation with stereotactic radiosurgery, two factors associated with prolonged survival, and following an optimally aggressive approach to brain metastasis in osteosarcoma [11,12,13,14]. Cabozantinib was chosen due to reported antitumor activity in patients with advanced osteosarcoma [15] and reasonable evidence of CNS penetration, and nivolumab was added in an attempt to provide synergy with radiation. Immune profiling studies utilizing fixed tissue (Figure 3C–F) and freshly isolated single cell suspensions (Figure 3G,H) of brain tumor specimens pre- and post-therapy with cabozantinib and nivolumab exhibited changes in the immune milieu of the tumor microenvironment similar to those demonstrated in other malignancies in response to checkpoint inhibitors [16]. These changes may indicate an anti-tumoral immune response elicited by the intervention, potentially halting progression for 7 months; however, a definite histologic response to the immunotherapy was not identified. This observation addresses the importance of considering patterns of immune infiltrates in the tumor microenvironment as markers of response to therapy in addition to traditional methods used to assess tumor response, such as measuring necrosis, since they might not adequately capture the complexity of responses seen with immunotherapy. The use of cabozantinib and nivolumab in multimodal treatment regimens for patients with osteosarcoma and brain metastases, particularly combined with post-operative radiation, is reasonable to consider, and clinical trial data utilizing the combination of the TKI regorafenib and nivolumab in patients with osteosarcoma are expected to have results soon.

It has been suggested to screen patients with metastatic pulmonary osteosarcoma for intracranial disease [17], although no subsequent studies have confirmed the clinical utility of this practice. In our patient cohort, dedicated brain imaging in two patients with pulmonary disease failed to detect intracranial disease that later developed. Patient 6 underwent full body PET/CT without identification of intracranial disease 21 days prior to brain MRI, which detected 10 brain metastases measuring up to 3 cm. Patient 3 similarly had dedicated brain imaging three months before being diagnosed with intracranial disease. These anecdotes demonstrate that intracranial metastases may progress rapidly. We and others, however, have described [4] long-term disease control (greater than one year) with brain metastases. Therefore, it is essential to remain clinically vigilant with a low threshold to obtain imaging in patients with new-onset focal neurologic symptoms.

## 5. Conclusions

Taken together, this report advances our understanding of brain metastases in osteosarcoma by providing further descriptions of its natural history in six patients at a single institution. These outcomes highlight the well-established and critical role of surgery in managing osteosarcoma, as well as current diagnostic challenges for active surveillance. This study does, however, have limitations. We report a single institution retrospective experience, limited by a relatively small sample size. Hypothesis-driven prospective studies and clinical trials to identify potential treatments and to better understand the incidence, prognosis, and natural history of brain metastasis in osteosarcoma would be ideal but likely precluded by challenges to identification and enrollment of this rare population. Disease biology studies examining tumor features across primary, pulmonary, and brain metastatic sites offer an opportunity to better understand how this process develops in patients and identify therapies that may best target osteosarcoma brain metastases.

## Figures and Tables

**Table 1:** Clinical features of six patients with osteosarcoma who developed brain metastases.

Patient	Age/Sex	Initial Site	Metastasis at Initial Diagnosis	% Necrosis After Chemo	Months to Initial Progression	Months to Brain Metastasis	Overall Survival (Months)	Survival from Brain Metastasis (Months)
1	8/M	Femur	No	85	9.5	26.4	31.4	5
2	20/M	L5/Sacrum	No	0	5.6	7.7	8.1	0.4
3	16/M	Femur	Yes (lungs)	55	6.4	10.4	13.8	3.4
4	11/M	Tibia	Yes (lungs)	50	21.3	38.3	39.9	1.6
5	9/M	Femur	No	90	14.9	30.8	44	13.2
6	17/F	Femur	Yes (lungs)	15	3.7	8.6	11.3	2.6
Median:	13.5	-	-	52.5	7.95	18.2	22.6	2.5

## Data Availability

The data presented in this study are available on request from the corresponding author.
